# Analysis of single‐cell RNAseq identifies transitional states of T cells associated with hepatocellular carcinoma

**DOI:** 10.1002/ctm2.133

**Published:** 2020-07-13

**Authors:** Yanying Yang, Fangming Liu, Weiren Liu, Mingyue Ma, Jie Gao, Yan Lu, Li‐Hao Huang, Xiaoying Li, Yinghong Shi, Xiangdong Wang, Duojiao Wu

**Affiliations:** ^1^ Department of Endocrinology and Metabolism Zhongshan Hospital Key Laboratory of Metabolism and Molecular Medicine the Ministry of Education Fudan University Shanghai China; ^2^ Institute of Clinical Science Zhongshan Hospital Fudan University Shanghai China; ^3^ Liver Surgery Department of Zhongshan Hospital Fudan University Shanghai China; ^4^ Department of Pathology & Immunology Washington University School of Medicine Saint Louis Missouri

**Keywords:** CD8 T cell, exhaustion, hepatocellular carcinoma, regulatory T cell

## Abstract

**Background:**

Exhausted T cells and regulatory T cells (Tregs) comprise diverse subsets of tumor immunosuppressive microenvironment that play key roles in tumor progress. Understanding subset diversity in T cells is a critical question for developing cancer immunotherapy.

**Methods:**

A total of 235 specimens from surgical resections of hepatocellular carcinoma (HCC) patients were examined for infiltration of exhausted T cell (Tex) in tumor and adjacent tissue. We conducted deep single‐cell targeted immune profiling on CD3^+^ cells collected from tumor tissues, adjacent normal tissues (ANTs) and peripheral blood of HCC patients. Total 10 cell clusters were identified with distinct distributions and characteristics.

**Results:**

We observed transitional differentiation of exhausted CD8^+^T cells and Tregs increasingly enriched in tumor tissue. The accumulation and location of Tex were related to the differences in the long‐term clinical outcome of HCC. Furthermore, data of single‐cell RNA‐seq showed that (1) cells transforming from effector CD8^+^ T cells to exhausted CD8^+^ T cells simultaneously expressed upregulated effector molecules and inhibitory receptors, (2) indicated alteration of gene expression related to stress response and cell cycle at early exhaustion stage, and (3) immunosuppressive Treg had profound activation in comparison to resting Tregs.

**Conclusions:**

T cell exhaustion is a progressive process, and the gene‐expression profiling displayed T cell exhaustion and anergy are different. Accordingly, it is possible that functional exhaustion is caused by the combination effects of passive defects and overactivation in stress response. The results help to understand the dynamic framework of T cells function in cancer which is important for designing rational cancer immunotherapies.

## Graphical abstract

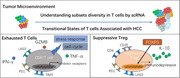

## 1 INTRODUCTION

Hepatocellular carcinoma (HCC), which accounts for 85% to 90% of the pathological types of primary liver cancer, has been rising globally in the past years, and now it is the fifth common malignant tumor that ranks the third leading cause of cancer‐related mortality evaluated by the World Health Organization (https://www.who.int/).^1^ It is expected that it will continually to increase in some countries including the United States until 2030.^2^

In recent years, immune checkpoint inhibitors, such as PD‐1 and CTLA‐4 inhibitors, have been indicated as promising treatments for patients with cancers.^3^, ^4^, ^5^ These “checkpoint blockade” therapeutics are aimed at improving tumor infiltrating T cells (TIL) activity by alleviating the regulatory mechanisms.^6^ Some antibodies, such as nivolumab (anti‐PD‐1), pembrolizumab (anti‐PD‐1), and ipilimumab (anti‐CTLA‐4), have been approved by the US FDA and have shown excellent therapeutic effect on melanoma, nonsmall cell lung cancer, glioblastoma, large B‐cell lymphoma, multiple myeloma, renal cell carcinoma, and so on.^7^, ^8^, ^9^, ^10^ Interestingly, when patients with advanced HCC, immune checkpoint inhibitors also provide beneficial results.^4^, ^11^, ^12^, ^13^ Although checkpoint blockade, such as PD‐1 inhibitors can improve clinical outcomes, the efficacy is variable between tumors or patients.^14^, ^15^ In clinical practice of the treatment of HCC, the immunotherapy is represented by PD‐1 or PD‐L1; CTLA‐4 antibodies had a low disease remission rate.^16^, ^17^, ^18^ The reason is that in addition to the formation mechanism of the immune tolerance microenvironment of liver cancer is complex, other factors related to chronic liver disease may also affect liver‐specific immune cells and the patient's response to this immunotherapy.^19^, ^20^, ^21^

One of possibilities to explain the variation could be due to the major barriers of tumor therapies from exhausted T cells and regulatory T cells (Tregs),^22^, ^23^ which have displayed a dramatically altered pattern of differentiation.^24^, ^25^ Therefore, it is essential to comprehend that T cell immune community, which tightly links to tumor immunotherapy response, in order to provide improved strategies to coordinate the immune response system to eradicate cancer. According to current knowledge and controversial/ inconsistent results, we set out a novel study using single‐cell transcriptome analysis from CD3^+^ cells to investigate cell transition or differentiation leading to terminal exhaustion or the accumulation of Tregs in HCC in a highly complex tumor biological context.

This approach revealed the progressive process of TIL differentiation and biology in HCC. First, the expression of various cell‐surface inhibitory receptors was elevated in Tex. Second, the transcription level of genes encoding molecules involved in stress response was downregulated and possibly weakened the efficiency of these pathways. Third, multiple genes related to cell cycle and growth factors were altered in the early exhausted CD8^+^ T cells. Fourth, a striking changed differentiation pattern was found in suppressive Treg cells, compared to resting Tregs, including unique expression patterns of transcription factors, cytokines and interleukins. It is suggested potential new targets of therapies in HCC by the defections and alternations of pathways confirmed in the exhausted CD8^+^ T cells or Tregs

## 2 METHODS

### 2.1 Human specimens

This study enrolled 248 patients with pathological diagnosis of HCC. Before the tumors were resected, all patients did not undergo chemotherapy or radiation therapy. Paired HCC tumors, ANTs, or peripheral blood were collected from patients. ANTs were more than or equal to 3cm away from the matched tumor tissues. Fresh specimens from 13 HCC patients were obtained for the subsequent CD3^+^ cell sorting, then single‐cell RNA sequencing analysis or in vitro testing. Ethics committee’ approval was obtained from Zhongshan Hospital, Fudan University, and written informed consent was obtained from all HCC patients.

### 2.2 Sample preparation

Ficoll solution (17544202 GE healthcare) was used to extract peripheral blood mononuclear cells (PBMCs) in accordance with the manufacturer's protocol. In short, 10 mL of fresh peripheral blood was harvested in an anticoagulation tube before surgery. The peripheral blood was diluted with PBS at 1:1 dilution and gently transferred to a centrifuge tube with Ficoll. PBMCs were isolated by fractionation over Ficoll gradients, and then washed twice with 1 × PBS.

Fresh tumor and ANT specimens were soaked in RPMI‐1640 medium (Gibco) with 10% FBS, then cut, triturated, and passed through 40 μm cell strainers. Next, the suspended cells were centrifuged at 1500 rpm for 10 min. After removing the supernatant, erythrocyte lysis buffer (Solarbio) was used to resuspend the cell pellets and incubated on ice for 5 min. The cell pellets were washed twice with 1 × PBS.

### 2.3 Tissue microarrays and multiplex quantitative immunofluorescence

A total of 235 formalin‐fixed paraffin‐embedded liver cancer samples were collected randomly from HCC patients at Zhongshan Hospital, Fudan University (Shanghai, China) during 2006 and 2007. As mentioned previously, tissue microarrays (TMAs) were conducted by Shanghai Biochip Co., Ltd. It was carried out according to the instruction by the multiplex quantitative immunofluorescence staining for TMAs slides. Slides were fluorescently stained with CD8, PD1, CD4, and FOXP3 antibodies by Opal 7‐Color Manual IHC Kit (NEL811001KT), and the Vectra Polaris multispectral imaging system (PerkinElmer) was used to acquire multispectral images of arrays. As reported before,^26^ after multilabeled immunofluorescence, cells were identified through the Cell Segmentation function of Inform software. Then Score function was used to calculate fluorescence intensity. A positive rate of cells refers to the fluorescence intensity of a single‐cell exceeded the threshold value. Positive rates of makers (such as CD8^+^, CD8^+^PD‐1^+^) in peritumor or tumor were divided into low and high expression groups according to a cutoff value of overall survival (OS) assessed by X‐tile 3.5.0.

### 2.4 FACS analysis

Cells were stained with CD4 (BioLegend, clone: RPA‐T4), CD3 (BioLegend, clone: HIT3a), CD8 (BioLegend, clone: SK1), PD‐1(Biolegend, clone:EH12.2H7), CD45RO (Biolegend, clone:UCHL1), CD45RA(BD Pharmingen, clone:HI100), T‐bet (BioLegend, clone: 4B10), Eomes (R&D, 644730), Blimp(BD, cline: 6D3), NFATC (Biolegend, clone: 7A6), PFR (Biolegend, clone: dG9), CD16 (BD Pharmingen,clone: B73.1), CD56 (BD Pharmingen, clone: MY31), CD25 (BD Pharmingen, clone: 2A3), CD127 (BD Pharmingen, clone: HIL‐7R‐M21), IFN‐γ (BD Pharmingen, clone: B27) conjugated to various fluorochromes. FACS analysis was conducted on a BD FACS Aria II flow cytometer, and FlowJo v10 was used for analysis.

### 2.5 Single‐cell RNA sequencing

CD3^+^ cells from blood and tissues were sorted by using the Human CD3 Microbeads Isolation Kit (Miltenyi Biotec, 130‐050‐101). Immune response of isolated cells was analyzed at the single‐cell level by the BD Rhapsody Single‐Cell Analysis System and BD Rhapsody™ Immune Response Panel (Human). A total of 6267 cells were sequenced (Table S1 in the Supporting Information). The amplification system and the library of the single‐cell mRNA transcriptome final sequencing were obtained by targeted amplification. The analysis process was started with the FASTQ files of paired between Read1 and Read2, and through multistep quality inspection filtering, sample marker retrieval, single‐cell expression profile matrix generation, single‐cell population cluster analysis, and finally was completed by single‐cell targeted sequencing. Every cell was detected 399 target genes associated with T cell function.

### 2.6 ATAC‐seq sample preparation, sequencing, and analysis

CD3^+^CD8^+^CD45RO^+^ cells were isolated from tumor or adjacent tissue of HCC patients by FACS (Aria II from BD). As mentioned previously,^27^ the ATAC‐seq libraries were prepared using 50,000 cells from each sample. In brief, cells were suspended in 50 μL lysis buffer (0.1% (v/v) IGEPAL® CA‐630, 10 mM Tris–HCl (Ph 7.4), 3 mM MgCl_2_, 10 mM NaCl), and washed with ice‐cold PBS. The nuclei pellets were incubated in 50 μL tagmentation mix: 2× reaction buffer and 2.5 μL Tn5 Transposase (Nextera kit, illumina) at 37°C for 30 min. The Qiagen MinElute PCR purification kit was used to purify this labeled DNA, and the ATAC‐seq libraries were prepared by the NEBNext Ultra DNA kit, and sequenced with illumina Hiseq2000 using 75 bp paired‐end reads after size selection.

Reads were trimmed and mapped into items of the reference human genome (UCSC hg38) using Bowtie 2, then filtered on quality, removing duplicates and reads mapped to the M chromosome using SAM tools and Picard tools. For downstream analysis, we normalized the reading counts to 1× depth (reads per genome coverage, RPGC) by the “bamCoverage” function of deep Tools.^28^ The peak calling was conducted by “callpeak” function of MACS2 with the following parameters: –nomodel –shift ‐100 –extsize 200 –q 0.05. The signal tracks were visualized by the UCSC genome browser.

### 2.7 Statistical analysis of in vitro tests

Statistical analysis was conducted with prism 5 software (Graph pad). Paired or unpaired two‐tailed Student's *t* tests were carried out on comparisons of two groups. Contingency table analysis and χ2 tests were utilized to examine the relationship between clinical data and multilabeled immunofluorescence data of TMAs. As reported before,^26^ we calculated positivity of CD8^+^, CD8^+^PD‐1^+^ cells in duplicate for each dot. Then, the OS cutpoint was judged according to X‐tile 3.5.0, and the positivity of CD8^+^, CD8^+^PD‐1^+^ cells from tumor or normal tissues. TMA was divided into low or high expression group. The chi‐square test was used for statistical analysis, and statistically significant was defined *P* values of < .05. So as to research on survival or recurrence rates, Kaplan‐Meier estimates were used to calculate and plot time to recurrence (TTR) curves and OS with GraphPad Prism 5. The basis for TTR grouping and the aforementioned OS statistics were the same. All data of life tables were analyzed using the statistical package SPSS to investigate 1‐, 3‐, and 5‐year OS and recurrence rates. COX regression analysis was conducted for univariate and multivariate analysis of hazard ratio using SPSS statistics.

## 3 RESULTS

### 3.1 Clinical information and clinical relevance of Tex in HCC

We collected 235 HCC patients’ tissue array and summarized their clinical information in Table 1. All patients have more than 5 years of follow‐up. Through univariate and multivariate analysis, 15 key clinicopathological features were calculated to evaluate their relevance of the time to relapse (TTR) and the OS in HCC. The infiltrating Tex presented in the tumor core (TM) or ANTs were determined by multiplex quantitative immunofluorescence staining of PD‐1, CD8, and DAPI.

**TABLE 1:** Clinical information of patients

	Number of patients			Number of patients		
Variable	CD8^+^PD‐1_P^low^	CD8^+^PD‐1_P^high^	*P* value	CD8^+^PD‐1_T^low^	CD8^+^PD‐1_T^high^	*P* value
Age						
≤50 yr	71	24	.66	21	74	.321
> 50 yr	101	39		39	101	
Sex						
Female	30	11	.997	14	27	.164
male	142	52		46	148	
HBsAg						
Negative	31	4	.026	7	28	.416
Positive	141	59		53	147	
TB						
≤17 μmol/L	116	50	.075	38	128	.15
> 17 μmol/L	56	13		22	47	
ALT						
≤70 U/L	148	48	.072	49	147	.675
> 70 U/L	24	15		11	28	
ALB						
≤3.5 g/dl	9	1	.22	3	7	.741
> 3.5 g/dl	163	62		57	168	
Preoperative AFP						
≤20 ng/mL	60	23	.818	23	60	.571
> 20 ng/mL	112	40		37	115	
Liver cirrhosis						
No	25	15	.094	8	32	.378
Yes	147	48		52	143	
Tumor size						
≤5 cm	107	39	.966	41	105	.251
> 5 cm	65	24		19	70	
Multinodular tumor						
Single	144	53	.94	53	144	.272
Multiple	28	10		7	31	
Edmondson‐Steiner						
I‐II	131	46	.62	46	131	.779
III‐IV	41	17		14	44	
γ‐GT						
≤54	92	36	.618	39	89	.058
>54	80	27		21	86	
BCLC stage						
A	97	38	0.59	38	97	0.285
B+C	75	25		22	78	
Vascular invasion						
Absent	110	45	.284	38	117	.619
Present	62	18		22	58	
Tumor encapsulation						
Complete	91	34	.885	33	92	.745
None	81	29		27	83	

A I‐value < .05 was considered statistically significant. P‐values were calculated using the Pearson chi‐square test.

Abbreviations: AFP, α‐fetoprotein; ALB, albumin; ALT, Alanine aminotransferase; BCLC stage, Barcelona clinic liver cancer stage; γ‐GT, γ‐glutamyl; HBsAg, hepatitis B surface antigen; TB, total bilirubin.

**FIGURE 1:**
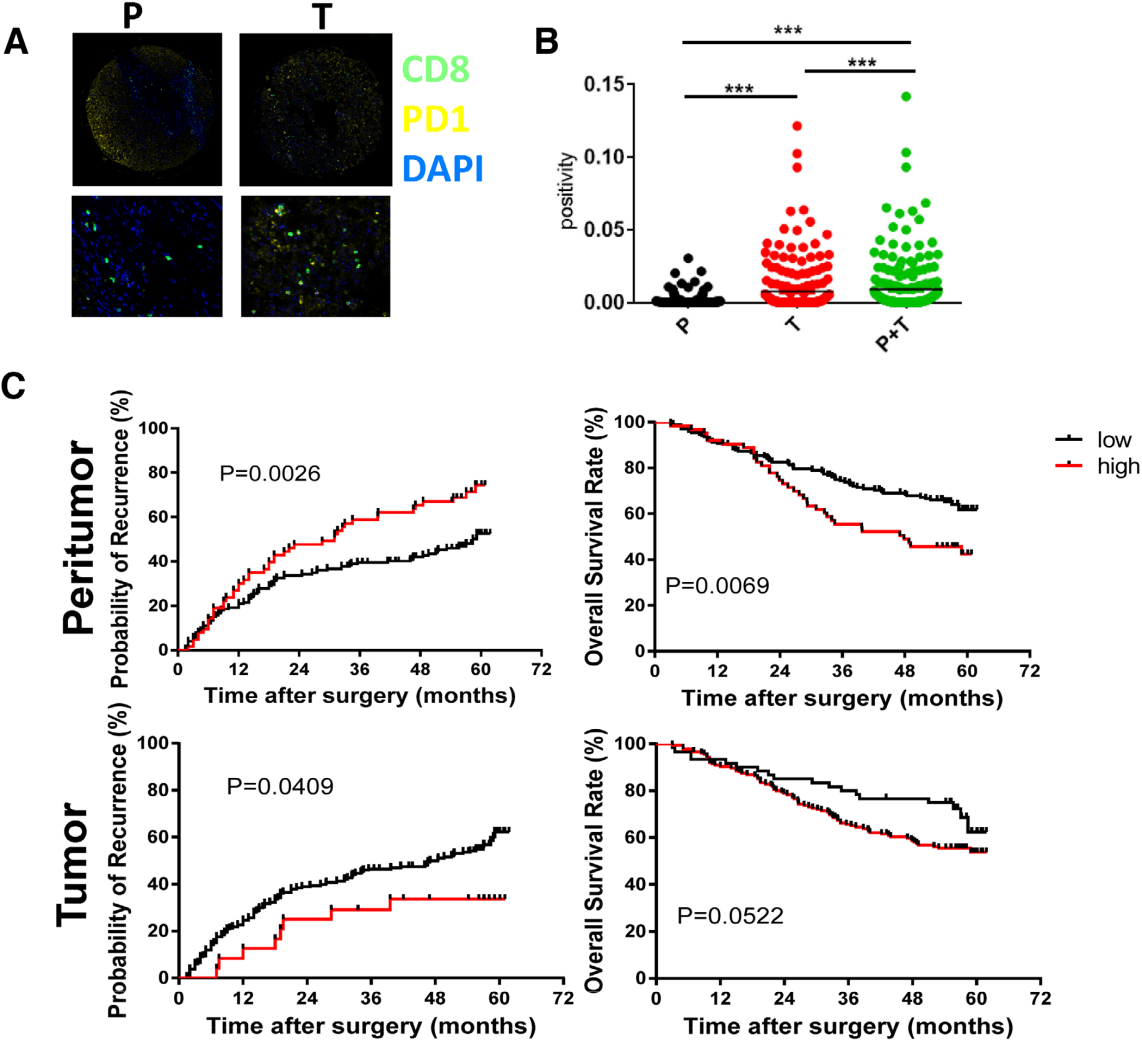
Clinical relevance of Tex in HCC. (A) Opal multicolor IHC staining with anti‐CD8, anti‐PD‐1, antibodies, and DAPI. We developed tissue array of 235 HCC patients and summarized clinical characteristics in Table 1. Each patient has more than 5‐year follow‐up. (B) Comparison of CD8^+^PD‐1^+^ T cells in tumor or peritumor. (C) Clinical relevance of Tex in HCC. TTR and OS curves were plotted in GraphPad Prism . The horizontal axis showed time after surgery and the vertical axis showed probability of recurrence or OS rates by the corresponding time. Two sets of data (low vs high) were represented by black and red curve, respectively.^low^, low expression of indicated biomarkers;^high^, high expression of indicated biomarkers. TTR, time to recurrence; DAPI: 4′,6‐diamidino‐2‐phenylindole; HCC: hepatocellular carcinoma; OS: overall survival; PD‐1: programmed death‐1; *P < .5; **P < .01; ***P < .001

Double positive of PD1 and CD8 markers was used to define the exhaustion status. More Tex and Treg accumulated in tumor (Figure 1A, B; Supplemental Figure 1a and 1b). We divided all samples into low or high group according to their PD1 expression. Then the association between the PD1 expression of CD8^+^T cells and 15 clinical measurements was analyzed. The data of Table 1 showed that in peritumor tissue, positive HBsAg infection significantly correlated with higher PD1 expression (*P* = .026). Combing peritumor and tumor staining together, advanced tumor stage (Edmondson‐Steiner) predicts enhanced PD1 expression in CD8^+^T cells (*P* = .018, data not shown). Then we found the location of Tex is significant clinically relevant in HCC. Fewer Tex in peritumor predicts higher survival rates of patients and lower recurrence (Figure 1C). Meanwhile, in tumor, fewer Tex predicts increased recurrence probability (Figure 1C). Taken together, the data suggest that first the accumulation of Tex is tightly related with clinical outcome of HCC and second the location of Tex has different prediction values.

### 3.2 ScRNA and clustering analysis

**FIGURE 2:**
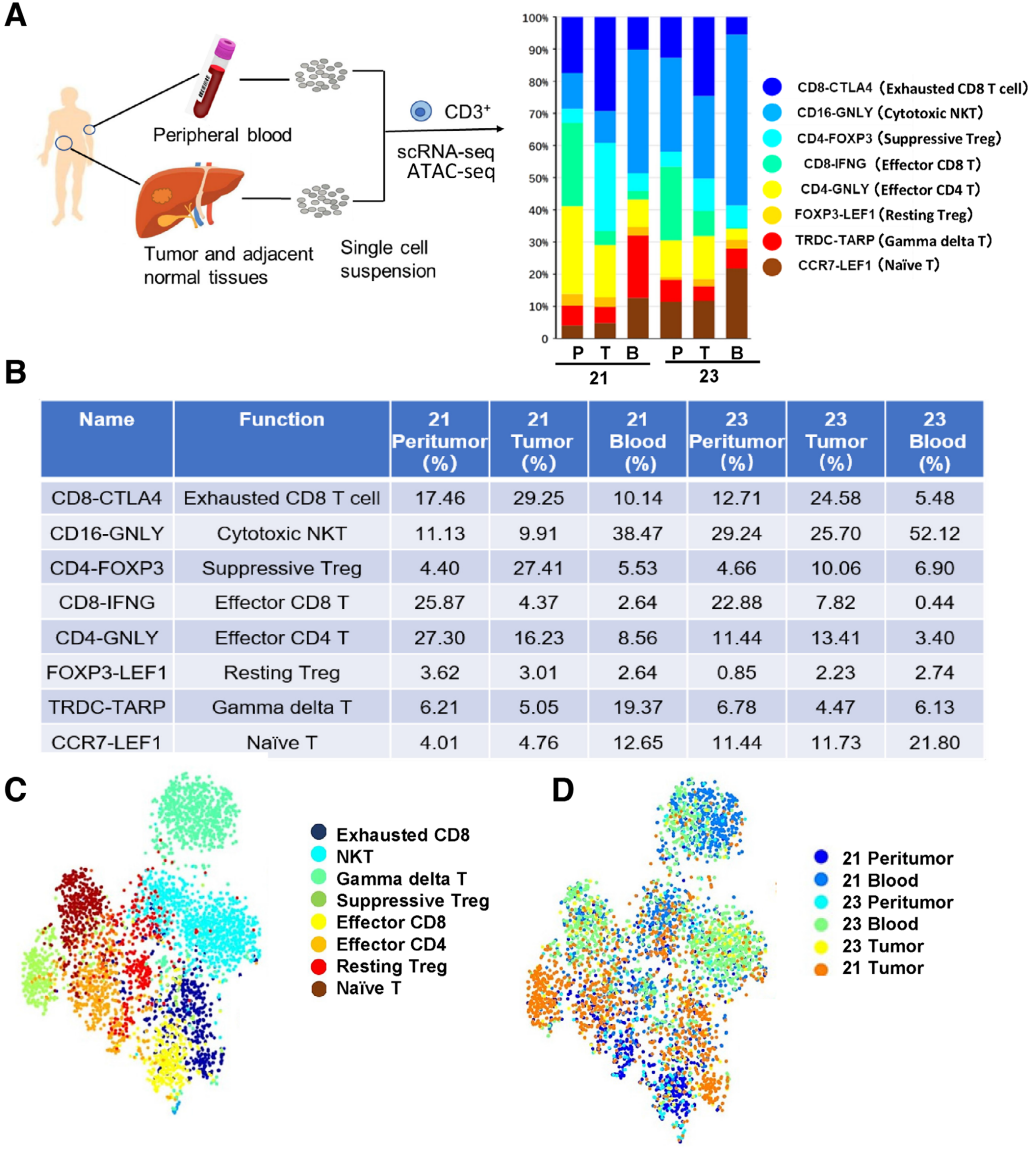
Single CD3^+^ cell transcriptome analysis. (A‐B), Scheme of the overall study design. Single‐cell RNA sequencing was applied to T cells derived from two patients’ (no. 21 and no. 22) peripheral blood (B), tumor (T), and adjacent normal tissues (P). Paired peritumor tissue or peripheral blood were obtained from same patients. The adjacent normal tissues were at least 3 cm from the matched tumor tissues. Histogram showing the average percentage of different types of T cells in different tissues. This table quantified the proportion. (C) T‐SNE visualization of T cells clusters based on 6267 single‐cell transcriptomes. (D) The t‐SNE projection of two patients, showing the formation of cell origin

The clinical characteristics highlight critical and predictive values of peritumor or tumor Tex; it suggests the importance of exploring the diversity of immune cells in HCC. Therefore, we performed deep scRNA on CD3^+^ cells isolated from tumors, adjacent normal tissues, and peripheral blood from HCC patients. The process is shown in Figure 2A. A total of 6267 cells from two patients (no. 21 and no. 22) was sequenced to detect lower expressed transcription factors and cytokines. The list of immune genes detected in the study is provided in Table S1 in the Supporting Information.

To illustrate the unique construction and underlying functional subgroups of the entire CD3^+^ cell population, unsupervised clustering of total CD3^+^ cells were carried out by the spectral clustering method. Altogether, we identified 10 stable cell clusters, including exhausted T cells, natural killer cells (NKs), gamma delta T, Treg cells, effector CD8^+^T cells, effector CD4^+^T cells, naïve T cells, monocyte and dendritic cells (DCs), granulocytes, etc. Since the proportion of monocyte and DCs, granulocytes were negligible due to CD3^+^ beads sorting, we excluded them in the further analysis. Exhausted CD8^+^T cells were dominant in tumor tissues and accounted for 29.25% and 24.58% of CD3^+^ cells (Figure 2B). At the same time, Treg cells accounted for 27.41% and 10.06% of CD3^+^ cells in tumor tissues, higher than ANT and blood (Figure 2B). Meanwhile, NKT, effector CD8^+^ or CD4^+^T cells predominantly existed in blood and peritumor tissues, respectively (Figure 2B). The distribution of clusters in the cell pool is shown in Figure 2C. Also we compared the aggregation of cells from different origins (blood, peritumor, and tumor; Figure 2D). The data indicated that the aggregation was shifted between patients (Figure 2D). For patient no. 21, cells from blood, peritumor, or tumor were clearly separated. However, for patient no. 23, the borderline was blurred, which suggested the obvious heterogeneity between HCC patients. To verify these clusters, we exhibited representative FACS plots of Naïve T, NKT, Treg, CD4^+^Teff, CD8^+^ Teff, and CD8^+^ Tex in Supplemental Figures 2 and 3.

**FIGURE 3:**
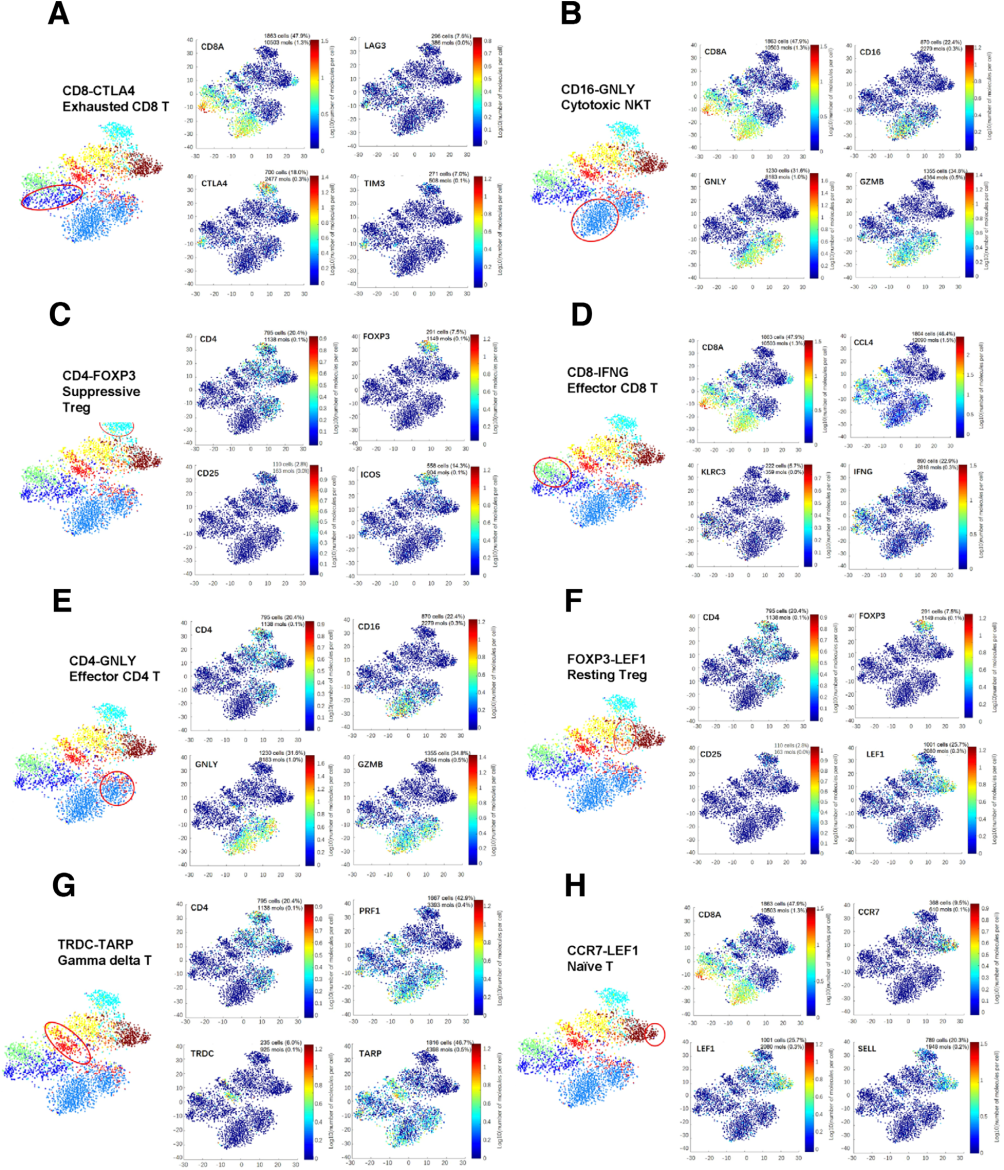
T‐SNE maps displaying representative markers in each cluster. (A‐H) t‐SNE maps displaying four representative markers in each cluster. The clusters include exhausted CD8^+^ T cell (exhausted CD8), natural killer cell (NK), gamma delta T, Treg cell, effector CD8^+^T cell, effector CD4^+^T cell, and naïve T cell

The representative markers in each cluster are shown in Figure 3. The first cluster is exhausted T cells, except PDCD1 (not shown), also expressing upregulated exhaustion markers such as TIM3, LAG3, and CTLA4 (Figure 3A). The second cluster, natural killer T cells (NKT), high expresses characteristic genes such as GNLY, CD16, and GZMB (Figure 3B), demonstrating their natural killing activity and effector functions. The third cluster is Tregs (Figure 3C), which play a role in suppressing immunity in tumor immunity. It is significantly higher in tumor tissues than in ANT and PBMC, expressing FOXP3, CD25, ICOS, and so on. The fourth cluster, cytotoxic CD8^+^ T cells (Figure 3D), was significantly decreased in tumors, which were confirmed by gene expressions including CCL4, IFNG, and KLRC3. Figures 3E‐H exhibited clusters (effector CD4^+^ cells, resting Treg, gamma delta T cells, naive T cells) and their expressions of representative markers.

### 3.3 Transitional exhausted CD8^+^ T cells

The results above illustrated that suppressive Tregs and Tex were preferentially enriched in tumor tissues. Compared with other T cells, we identified a total of 65 genes (adjusted *P* value < .05, and fold change > 2) that were specifically expressed in tumor Tex cells, including PI3, MKI67, UBE2C, TOP2A, IGLC3, TYMS, HMMR, KIAA0101, CD38, CHI3L2, etc. The top‐ranked genes were multiple known exhaustion markers, such as LAG3, HAVCR2, and PDCD1. Notably, some genes related to exhaustion were also overexpressed in tumor‐infiltrating Tregs including TYMS, KIAA0101, CXCL13, CD27, HLA‐DQB1, HLA‐DMA, ENTPD1, CD200, DUSP4, and ZBED2.

**FIGURE 4:**
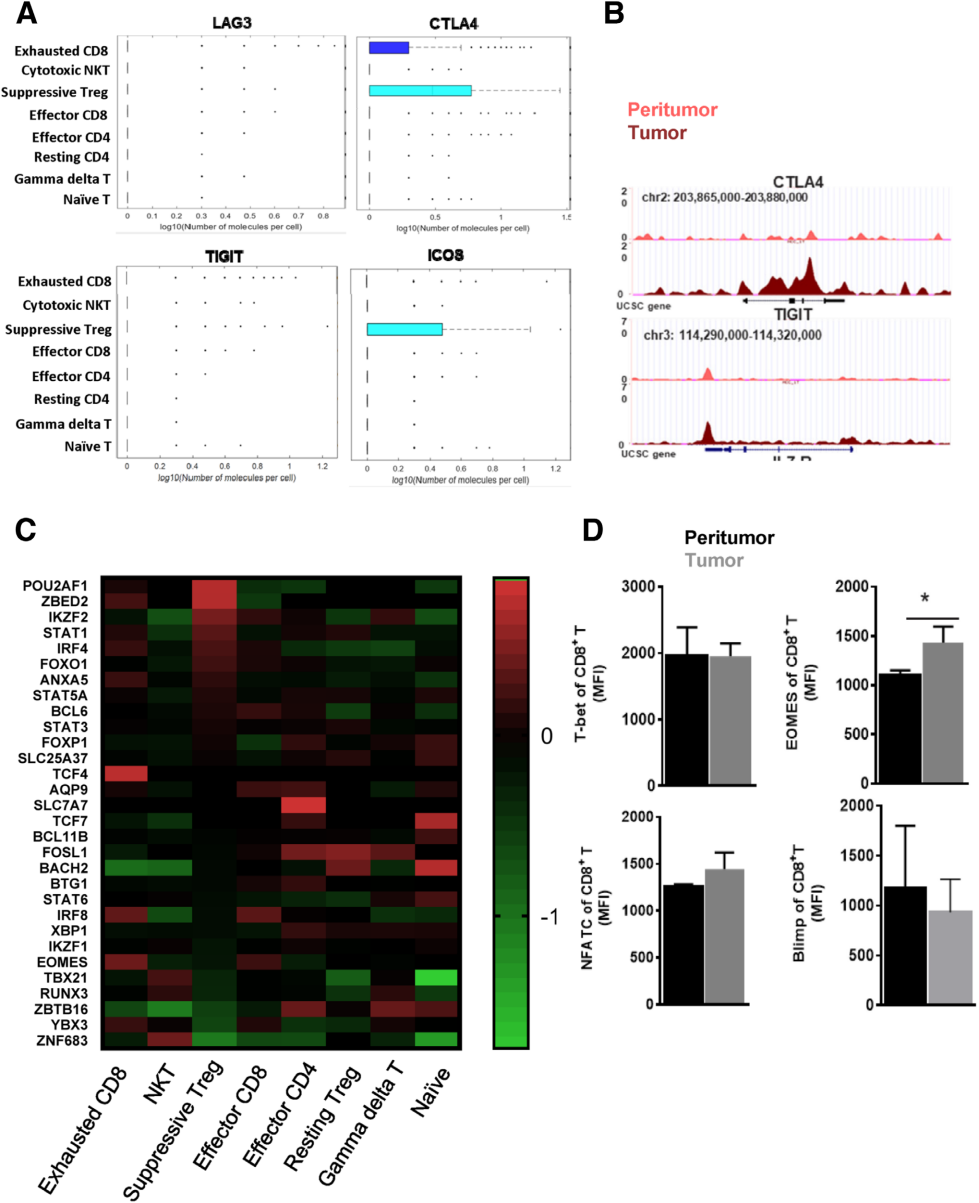
Characteristic of exhausted CD8^+^ T cells. (A) Dot plots showed the gene expression frequency made with BD's data view software. (B) The exhibition of accessible OCRs based on peak annotation with high throughput sequencing (ATAC‐seq) data of sorted CD3^+^CD8^+^CD45RO^+^ T cells between peritumor and tumor. Paired peritumor tissue and tumor tissue were obtained from same patients. The adjacent normal tissues were at least 3 cm from the matched tumor tissues. (C) Different transcription factors (TFs) expression patterns across different clusters. (D) The expression of these TFs at the protein level in CD3^+^CD8^+^CD45RO^+^T cells from tumor and peritumor by flow cytometry. The sample were obtained as described in Figure 4B

The two CD8^+^T cell clusters (CD8‐CTLA4, CD8‐IFNG) have distinct distributions, respectively, representing Tex and effector CD8^+^T cells. Exhausted CD8^+^T cells were found to be enriched in tumor, whereas effector CD8^+^T cells were the major group located in peritumor (Figure 2B). Tex specifically overexpressed multiple coinhibitory factors such as CTLA4 and ICOS (Figure 4A). We exhibited top well‐recognized exhaustion genes in Figure 4A. Also we analyzed the PD1 staining in a tissue microarray of 235 HCC patients as shown in Figure 1A. The data showed that CD8^+^PD1^+^T cells significantly accumulated in tumor than them in peritumor (Figure 1B). Next, we believe these genes that were uniquely regulated in T cells also exhibited specific epigenetic changes, which would provide more robust and stable signature of exhaustion. To verify this hypothesis, we identified enhancers in exhausted CD8^+^T cells from HCC by epigenomic profiling by assay for transposase‐accessible chromatin with high throughput sequencing (ATAC‐seq). Over 4662

differentially accessible genes based on the peak annotation were identified in ATAC‐seq data of sorted CD3^+^CD8^+^CD45RO^+^ T cells between peritumor and tumor, showing substantial chromatin remodeling. For example, *TIGIT*, *CTLA4* had more accessible open chromatin regions (OCRs) in tumors (Figure 4B). Therefore, some differentially expressed exhaustion‐specific genes, as identified in our scRNA analysis, were also associated with epigenetic changes.

We next sought to explore the expression of genes encoding key transcription factors (TFs) that enable T cell development. The heatmap revealed different TFs expression patterns across these clusters (Figure 4C). Tex and effector CD8^+^T cells shared similar TFs, but Tex expressed more TCF4. In comparison to naïve T cells or resting Tregs, suppressive Tregs (CD4‐FOXP4) exhibited distinct TFs expressions. We then examined the protein level of these TFs and found that Eomes was in fact differentially expressed in CD3^+^CD8^+^CD45RO^+^T cells from tumor and peritumor (Figure 4D, *P* < .05), with increased expression in the tumor.

**FIGURE 5:**
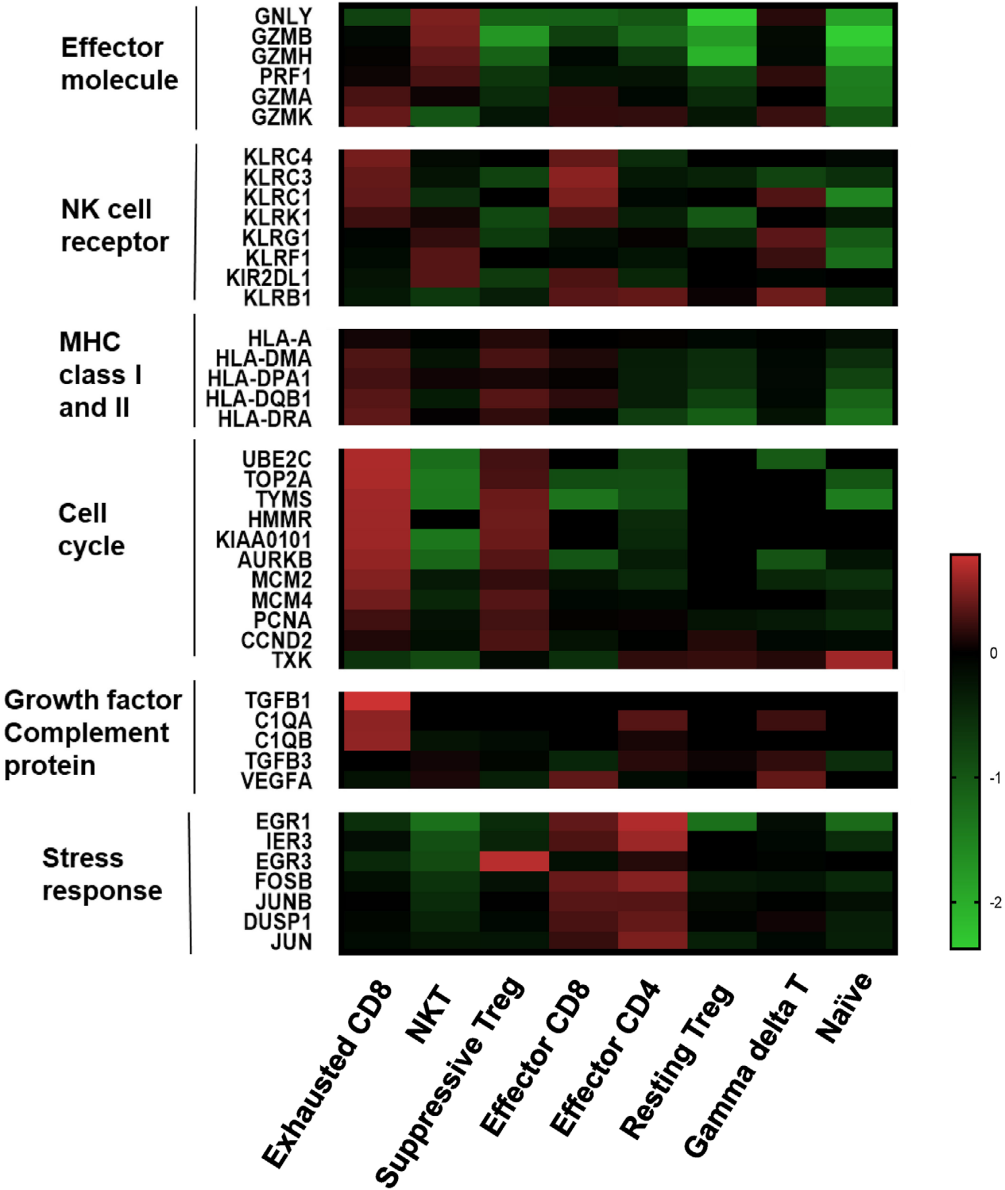
Comparison of key features between clusters. Heat map showing normalized mean expression of selected T cell function‐associated genes from scRNA in each cell cluster. It included critical genes coding effector molecule, NK cell receptor, MHC class I and II, cell cycle growth factor complement protein and stress response

Enhanced Eomes expression may imply that tumor had an “advanced exhaustion” phenotype, where cytokine production is impaired, and poor proliferative potential is observed despite partial cytotoxicity ability.^29^ However, we found both CD8 clusters expressed high levels of effector genes including *GZMA* and *GZMK* (Figure 5), therefore representing their effector CD8^+^T characters. However, cell cycle, growth factor‐related genes were upregulated in Tex. In contrast, stress response was downregulated in Tex. The difference indicates that CD8^+^ T cell exhaustion suggests the specific status of gene expression compared with that of naive, effector CD8^+^ T cells. The “CD8‐CTLA4” is a cluster of CD8^+^T cells during the transitional state from activation to exhaustion at an early stage, as seen by maintaining effector functions although some exhaustion genes were expressed (Figure 5).

### 3.4 Transitional regulatory CD4^+^T cells

CD4‐FOXP3 (suppressive Tregs) and FOXP3‐LEF1 (resting Tregs, rTregs) shared Treg signature genes (FOXP3, CD4). RTregs were CD45RA^+^Foxp3^lo^CD4^+^; suppressive or activated Tregs were CD45RA^−^Foxp3^high^CD4^+^.^30^, ^31^ They were present with location preferences. Suppressive Tregs were accumulated in tumor, but FOXP3‐LEF1 T cells were evenly distributed in peritumor, tumor, and blood (Figure 2B). The increased population of suppressive Tregs in tumor and the widespread expression of coinhibitory receptors (Figure 2B) potentially supported the immune‐suppressive nature of the tumor microenvironment.

**FIGURE 6:**
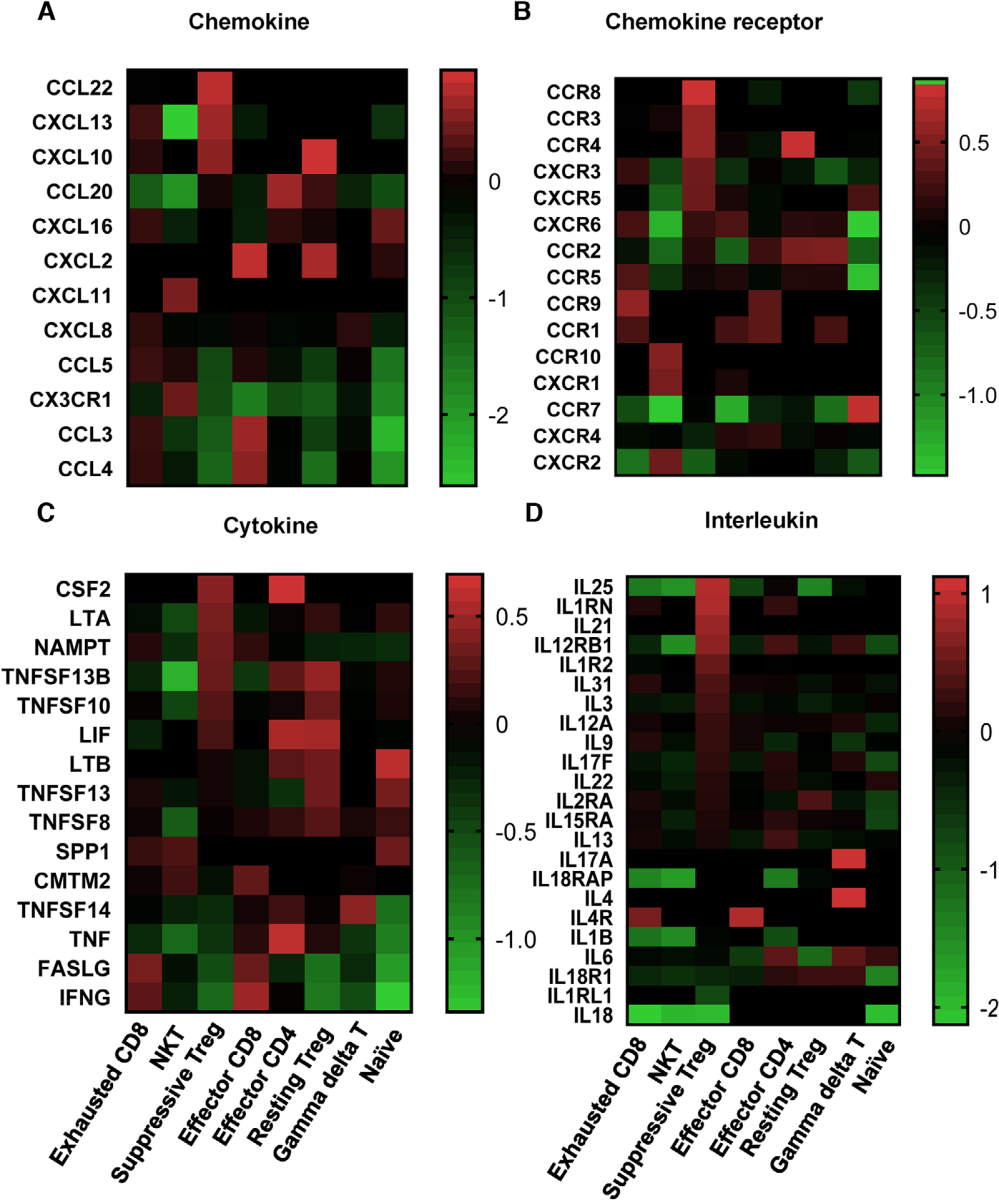
Heat map of differentially expressed genes coding chemokines, chemokine receptors, cytokines, and interleukins in each cluster. GraphPad Prism 5 was used to generate the heat map for exhibiting normalized mean expression of these genes from two patients’ scRNA

RTregs, when stimulated, can increase Foxp3 expression, convert to suppressive Tregs, and proliferate.^8^, ^31^ In Figure 6, the differentiation pattern of suppressive Treg cells was significantly changed compared with rTreg, including a distinct expression pattern of TFs, chemokines, chemokine receptors, cytokines, and interleukins (Figures 5 and 6). This reflects heterogeneity and gradual development of regulatory T cell development. The altered pathways identified in resting Treg or suppressive Tregs suggested potential targets for of therapeutic intervention in HCC.

## 4 DISCUSSION

Exhausted CD8^+^T cells and Tregs significantly affect tumor progress and efficiency of cancer immunotherapy.^32^, ^33^ Here, we investigated immunity in HCC patients with emphasis on the profiling of T cell subjects through deep single‐cell targeted immune profiling on CD3^+^ cells. We discovered for the first time that there are 10 clusters with distinct distributions and characteristics in HCC. We observed increased accumulation of Tex and Tregs in tumors. We further discussed changes related to the differentiation of Tex and Tregs. Understanding the altered pathways will help in designing new strategy for intervening progressive immunosuppressive microenvironment in HCC.

Exhausted T cells could be reinvigorated by the blockade of PD‐1, other inhibitory receptors, or immunoregulatory pathways.^14^, ^34^, ^35^ As we reported before, exhaustion is a progressive process in which cells over time express one or multiple inhibitory receptors. Multiple markers (LAG3, TIM3, CTLA4, PD1, etc.) were coexpressed in exhausted T cells of HCC. These therapies assume that exhausted T cells might be reversed, or these cells are not terminally exhausted and can contribute to protective immunity if reinvigorated.^36^ In the study, interestingly, we found that the location of Tex accumulation is differentially related to clinical outcome of HCC patients. Fewer Tex in normal tissues estimates higher survival rates and lower recurrence of patients. (Figure 1C). In contrast, in tumor, fewer Tex predicts increased recurrence probability (Figure 1C). The data suggest that heterogeneity of Tex between intratumor or peritumor. The different functions and states of Tex determine the distinct clinical relevance.

Therefore, defining the heterogeneity and abundance of early or terminal exhausted T cells becomes extremely important as a predictor of the check‐point blockade treatment. From scRNA data, we observed transitional differentiation of Tex were increasingly enriched in intratumor tissue. We found that the specific cluster Eomes^+^ cluster that was considered as a phenotype of terminal CD8 exhaustion subset showed diminished effector function.^37^, ^38^ However, genes that indicate effector functions, such as IFNG, GZMK, and GZMA, were retained in this subset (Figure 5). Therefore, we think Eomes is not reliable marker for the terminal exhaustion. Because the distinct tumor microenvironments are likely to influence T cell biology, we proposed that the definition of exhausted T cell is high variable in different diseases or locations. On the other hand, ATAC‐seq revealed some immune cosuppressor molecules in tumor T cells and displayed a more opened chromatin structure, suggesting potential regulations of exhausted T cell formation as reported before.^32^, ^39^

In Figure 5, cells displayed altered gene expression related to cell cycle and stress response at early exhaustion stage. Activated T cells included cytotoxic T cells and helper T cells, which mediated cellular immune responses by upregulating stress response genes as shown in Figure 5. Interestingly, signal transduction of cell death (EGR3, EGR1, JUN, DUSP1, JUNB, IER3) especially regulating early growth responses is suppressed in exhausted CD8^+^T cells. Thus, functional exhaustion is probably caused by the combination effects of passive defects and overactivation in stress response. Therefore, T cell exhaustion was a progressive process, and the gene expression profiles demonstrated T cell exhaustion and anergy are distinct.

Treg cells, a specialized lineage of suppressive CD4^+^ T cells, play a vital role in immune‐tolerance in various biological contexts.^40^ Treg cells exposed to inflammatory or tumor conditions obtain strongly enhanced suppressive function.^41^ Effector CD4^+^, resting Treg, and suppressive Treg cells are transcriptionally different (Figure 4C); Tumor‐infiltrating Treg cells have many activation‐induced changes including upregulated expression of chemokines, chemokine receptors, cytokines, and interleukins (Figure 6). We observed interleukin‐10 (IL‐10) signaling pathway (CSF2, CXCL10, IL1RN, ILR2, CCL22) is overactivated in suppressive Treg. IL‐10 is an anti‐inflammatory cytokine with important immunoregulatory functions.^42^ It is a cytokine with effective anti‐inflammatory properties, repressing Th17 cell‐mediated inflammation, and inflammatory cytokines production such as TNF‐α and IL‐6.^43^ Immunosuppressive Tregs have profound activation in comparison to resting Treg.

In summary, our study highlights transitional states of T cells associated with HCC. The results indicate the essential significance of immune status assessment in the context of tumor biology context. Current studies about the mechanisms of T cell exhaustion have provided some new perspectives as well as clinical directions and opportunities.^44^ In spite of the current progress, our research remains incomplete on the mechanism of T cell exhaustion and how to reverse this state more effectively. This study has several limitations. First of all, in the workflow of scRNA analysis, clustering is crucial for drawing conclusions. For cell type annotation, errors would be introduced into this process by the lack of specific markers or inadequate clustering analysis. In the study, we used combined analysis of bioinformatics methods and biological knowledge. Although some genes were expressed in different types of cells, the expression levels were different. We determined the cell type by the expression of most significant differentially expressed genes in each cluster. Second, we need collect more samples to depict the heterogeneity of HCC immune microenvironment. Meanwhile, further functional experiments are required to verify the relationship between cell dynamic differentiation and function in the tumor microenvironment, as well as the relationship between differentiation of T cell and tumor progression, so as to find targets for tumor intervention. Further clinical researches and mechanistic studies are necessary to develop the next generation of immune‐based interventions against cancers and chronic infections. The study shows the dynamic framework of T cells function in cancer, especially for designing rational cancer immunotherapies.

## CONFLICT OF INTEREST

The authors declare no conflict of interest.

## Supporting information

Supporting Information

Supporting Information

## Data Availability

The data that support the findings of this study are available from the corresponding author upon reasonable request.
