# Supplementary material for: Analysis of single‐cell RNAseq identifies transitional states of T cells associated with hepatocellular carcinoma
Source: Clin Transl Med. 2020 Jul 13;10(3):e133. doi: 10.1002/ctm2.133 (PMC7418813; doi:10.1002/ctm2.133)
Supplement: Supplementary file 2 — Supporting Information [file CTM2-10-e133-s002.pptx]

## Slide 1
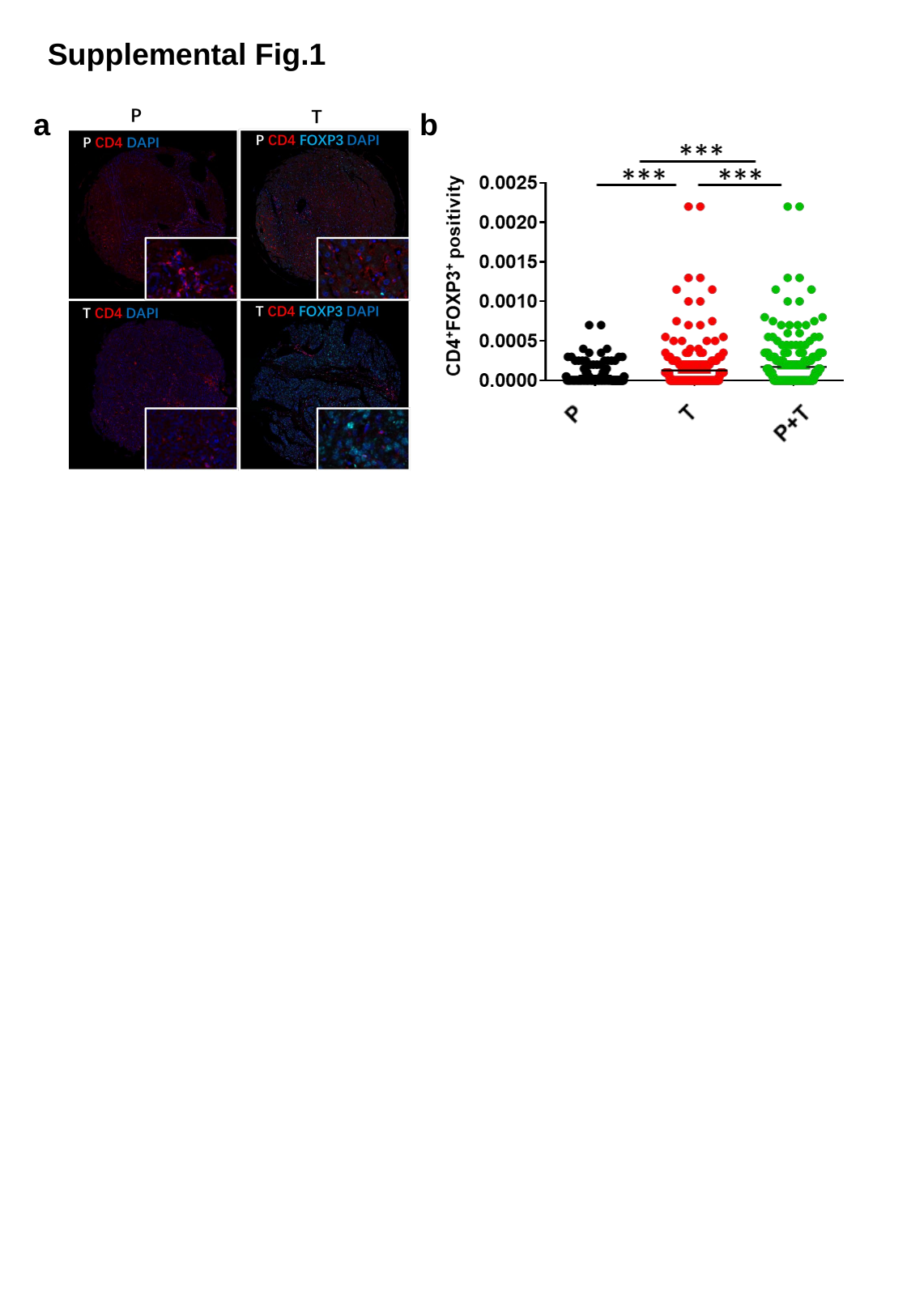

Supplemental Fig.1
b
a

## Slide 2
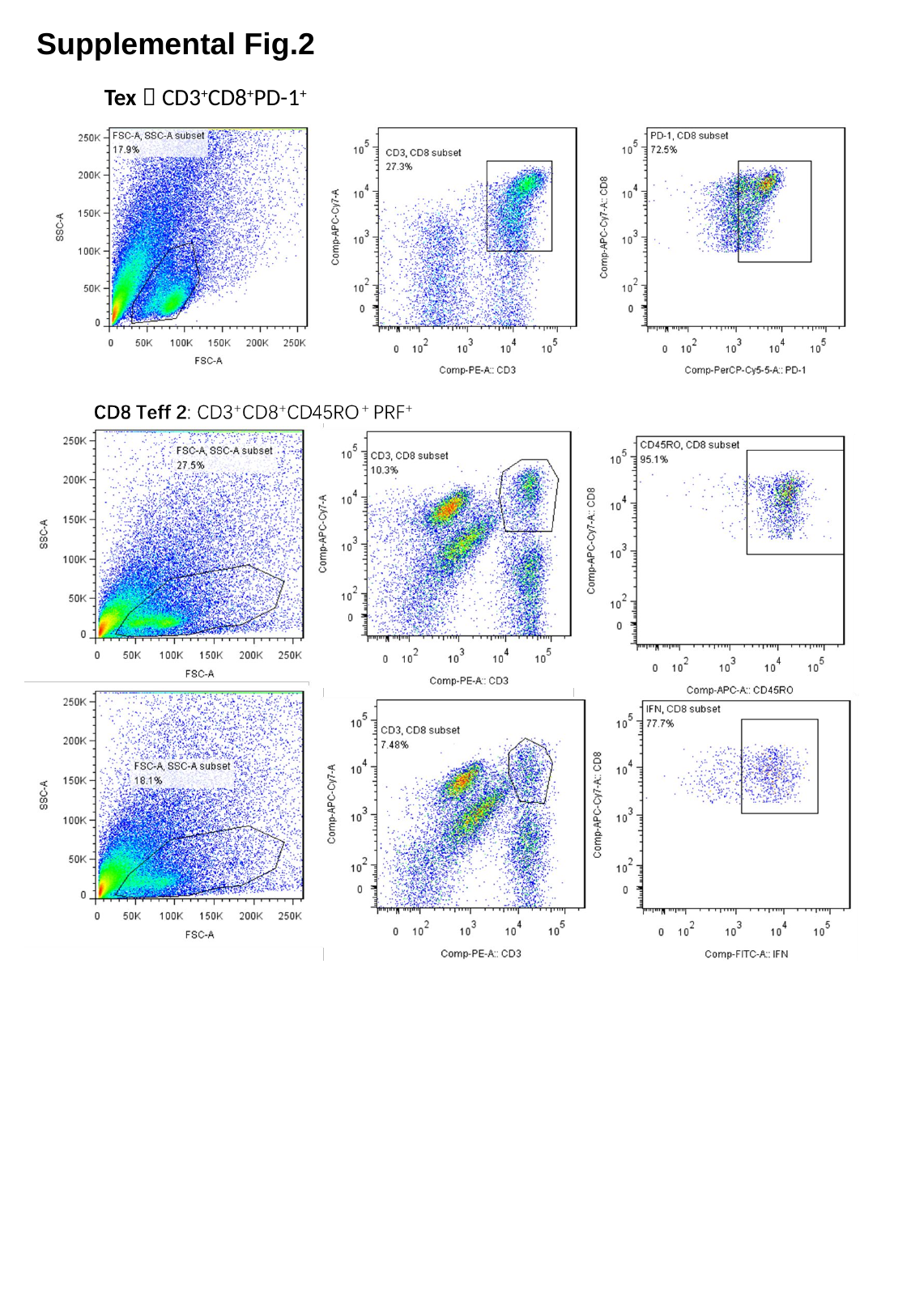

Supplemental Fig.2
Tex：CD3+CD8+PD-1+

## Slide 3
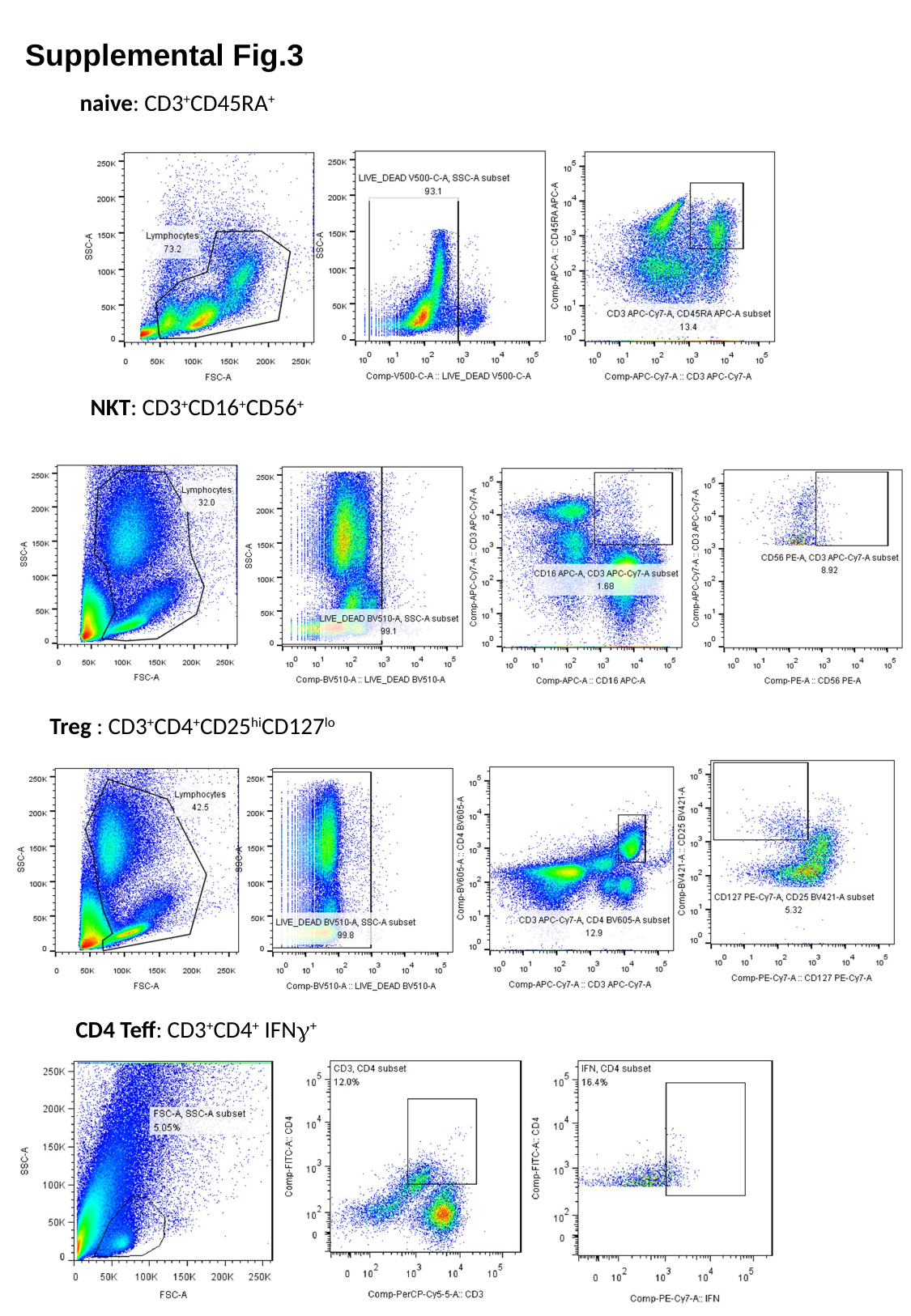

Supplemental Fig.3
naive: CD3+CD45RA+
NKT: CD3+CD16+CD56+
Treg : CD3+CD4+CD25hiCD127lo
CD4 Teff: CD3+CD4+ IFNg+
